# Application of Wavelet Packet Transform to detect genetic polymorphisms by the analysis of inter-Alu PCR patterns

**DOI:** 10.1186/1471-2105-11-593

**Published:** 2010-12-09

**Authors:** Maurizio Cardelli, Matteo Nicoli, Armando Bazzani, Claudio Franceschi

**Affiliations:** 1 Italian National Research Centers on Aging (I.N.R.C.A.), Ancona, Italy; 2 Dept. of Physics and Interdept. Center L.Galvani of Bologna University and INFN, Bologna, Italy; 3 Dept. of Experimental Pathology and Interdept. Center L.Galvani of Bologna University, Bologna, Italy

## Abstract

**Background:**

The analysis of Inter-Alu PCR patterns obtained from human genomic DNA samples is a promising technique for a simultaneous analysis of many genomic loci flanked by Alu repetitive sequences in order to detect the presence of genetic polymorphisms. Inter-Alu PCR products may be separated and analyzed by capillary electrophoresis using an automatic sequencer that generates a complex pattern of peaks. We propose an algorithmic method based on the Haar-Walsh Wavelet Packet Transformation (WPT) for an efficient detection of fingerprint-type patterns generated by PCR-based methodologies. We have tested our algorithmic approach on inter-Alu patterns obtained from the genomic DNA of three couples of monozygotic twins, expecting that the inter-Alu patterns of each twins couple will show differences due to unavoidable experimental variability. On the contrary the differences among samples of different twins are supposed to originate from genetic variability. Our goal is to automatically detect regions in the inter-Alu pattern likely associated to the presence of genetic polymorphisms.

**Results:**

We show that the WPT algorithm provides a reliable tool to identify sample to sample differences in complex peak patterns, reducing the possible errors and limits associated to a subjective evaluation. The redundant decomposition of the WPT algorithm allows for a procedure of best basis selection which maximizes the pattern differences at the lowest possible scale. Our analysis points out few classifying signal regions that could indicate the presence of possible genetic polymorphisms.

**Conclusions:**

The WPT algorithm based on the Haar-Walsh wavelet is an efficient tool for a non-supervised pattern classification of inter-ALU signals provided by a genetic analyzer, even if it was not possible to estimate the power and false positive rate due to the lacking of a suitable data base. The identification of non-reproducible peaks is usually accomplished comparing different experimental replicates of each sample. Moreover, we remark that, albeit we developed and optimized an algorithm able to analyze patterns obtained through inter-Alu PCR, the method is theoretically applicable to whatever fingerprint-type pattern obtained analyzing anonymous DNA fragments through capillary electrophoresis, and it could be usefully applied on a wide range of fingerprint-type methodologies.

## Background

Many analytical methodologies in modern genetics and biochemistry are based on the analysis of complex mixtures of oligonucleotides or oligopeptides, which are resolved as complex patterns of peaks or bands often referred as "fingerprint type" patterns. When the analysis is performed at the DNA or RNA level, fingerprint type patterns can be generated by gel or capillary electrophoresis of nucleic acid sequences produced by PCR (Polymerase Chain Reaction) -based techniques, such as Random Amplified Polymorphic DNA (RAPD) [1], Arbitrarily Primed PCR (AP-PCR) [2], Simple Sequence Repeat anchored Polymerase Chain Reaction amplification (SSR-PCR) [3], Differential Display Reverse Transcription (DDRT) PCR [4], AFLP [5], inter-Alu PCR [6]. All these methodologies allow for a screening of several (up to some hundreds) nucleic acid fragments that correspond to different loci, without making any a priori assumption about their exact sequence and genomic localization. The comparative analysis of patterns obtained in different samples reveals its utility in the most disparate fields of biological research: as examples we recall the identification of genes overexpressed in tumors [5], the identification of genetic variability at different levels (individuals, populations, species) [7-9] and the discovering of genomic loci associated with human longevity [10]. Among DNA fingerprinting techniques, inter-Alu PCR [6,11,12] is of particular interest, being characterized by the highest information level [13]. Alu repeat sequences are ubiquitously distributed in the human genome with more than one million elements [14]. A genomic DNA fragment can be amplified with a single Alu-specific primer when it is flanked by two Alu elements which have opposite orientation and a distance within few kilobases. A PCR reaction conducted with one ore more primers complementary to Alu sequences produces a multitude of anonymous DNA amplification products that can be revealed by electrophoretic separation. A typical inter-Alu pattern often shows inter-individual variability, due to genetic polymorphisms of different types: length variation of intervening sequences, de novo insertion of flanking Alu elements, deletions, translocations, and mutation of priming sites [13,15,16]. In general, this approach can be used for the initial detection of polymorphic loci involved in quantitative, multigenic traits [10,17] or of germline and somatic mutations [18,19] or of genetic alterations in cancer cells [20-23]. In a previous study [10], we developed a variant of inter-Alu PCR, which uses two different Alu-specific primers labeled with different fluorochromes in the same PCR reaction; the resulting PCR products can be analyzed by capillary electrophoresis and fluorescent detection on a PE/ABI Genetic Analyzer, and reported by the instrument as distinct fluorescence peaks; many of the peaks generated by this method are smaller than 1 Kb and, given that the frequency peaks of Alu elements in the human genome are centered at 0.1 Alu/kb and 1 Alu/kb [24], are likely to be obtained from the regions with highest density of Alu sequences [10,17]. In the inter-Alu PCR analysis, as well as in other fingerprint-type genomic analysis, the comparative evaluation of the analytical samples is usually done "by eye" by the operator, with the time-consumption and the possible errors associated with a subjective evaluation. These limitations prevent the application of these technique to large data sets and there is the necessity to develop computer-based analytical approaches, able to automate the comparative analysis of different samples and to provide better reliability and operative efficiency. We have elaborated and tested, in the present work, an algorithm based on the Wavelet Packet Transformation (WPT) aimed to detect fingerprint-type patterns generated by inter-Alu PCR. The WPT is an overcomplete multiscale analysis of the initial signal based on wavelet functions [25]. Starting from a signal of length 2*^N ^*the information is distributed on *N *× 2*^N ^*coefficients so that it is possible to apply an optimization procedure for classification problems and pattern recognition. In recent years the wavelet analysis has been largely applied to biological data sets, for very different purposes such as microarray data mining [26,27] and analysis of the genomic sequence [28-30]. In this paper we use the Best Basis algorithm to define different classes of signals. This method has been developed by Coifman and Wickerhauser [31] for the sismic signals classification and successively applied to feature extraction problems by Saito [32] that has proposed the Local Discriminant Basis algorithm. The classification is based on the hypothesis that the relevant signal information is well reproduced by a limited number of wavelet coefficients. To perform the WPT we have chosen the Haar basis that generates the Walsh packets [33]. We have tested the capability of the wavelet analysis to detect sample to sample differences in a fingerprint type pattern produced by the electrophoretic analysis of inter-Alu PCR products. The positions of electrophoretic peaks detected by the genetic analyzer was used to reconstruct the inter-Alu pattern using a standard Gaussian for each peak. We have applied the WPT algorithm to identify some regions in the electrophoretic patterns where a significant difference is detected among the signals obtained from three couples of homozygotic twins. The comparison of the patterns of members of the same couple of twins allowed to filter the intrinsic variability of experimental methodology, whilst those signals which varied only among different twins were possibly correlated to polymorphic loci. The characterization of the detected polymorphic loci requires further specific experiments.

## Methods

**Figure 1:**
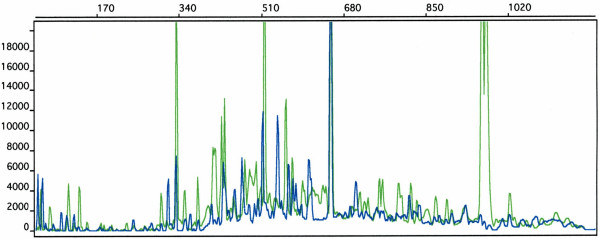
**Inter-Alu electrophoretic patterns**. Example of the electrophoretic inter-Alu pattern obtained from 310 Genetic Analyzer (PE/ABI) using the two primers, 5'-AGCGAGACTCCG-3' (R12A/267) labeled with the "TET" fluorochrome and 5'-CAGAGCGAGACTCT (R14B/264) labeled with the "FAM" fluorochrome: the *x*-axis units are base-pairs whereas the peak amplitude is in arbitrary units. Green peaks represent TET-labeled PCR products, while blue peaks represent FAM-labeled PCR products.

Whole blood DNA samples were obtained from three pairs of monozygotic twins, following standard procedures. All the subjects gave their informed consent. The recruitment of participants was carried out in compliance with the Helsinki Declaration, and after the approval of the Independent ethical committee of the Bologna Hospital-University was obtained. For each sample, four independent experimental replicates were performed, repeating the experimental procedure (inter-Alu PCR and electrophoresis) four times, in different days, in order to test the experimental variability. Details of inter-Alu PCR and capillary electrophoretic separation have been described in a previous article [10]. Briefly, inter-Alu PCR was conducted using two primers, 5'-AGCGAGACTCCG-3' (R12A/267) labeled with the "Tet" fluorochrome, and 5'-CAGAGCGAGACTCT (R14B/264) labeled with the "Fam" fluorochrome, using a 9700 thermal cycler (Applied Biosystems). Inter Alu-PCR products were then separated by capillary electrophoresis in a 310 Genetic Analyzer (PE/ABI). Each run was performed using Pop 4 (PE/ABI) denaturing electrophoresis polymer. Before running, 2*μl *of amplified samples were added to 12*μl *of formamide and to 0.8*μl *of a carboxytetramethylrhodamine-labeled internal size standard (Genescan 2500 size standard, supplied by PE/ABI). This program (supplied by PE/ABI) was used to visualize and to export the electrophoretic patterns (see Figure 1). The Gene-Scan program provides some relevant information: associated primer, peak position in data point (measure of the instrument), peak height and peak area. Repeated measures of the same sample indicated that the only reproducible entries were the peak position and the peak length (the ratio between area and height).

We have developed a program that performs the signals reconstruction using a mapping from data point (unit of the instrument) to base pairs.

### Noise reduction

**Figure 2:**
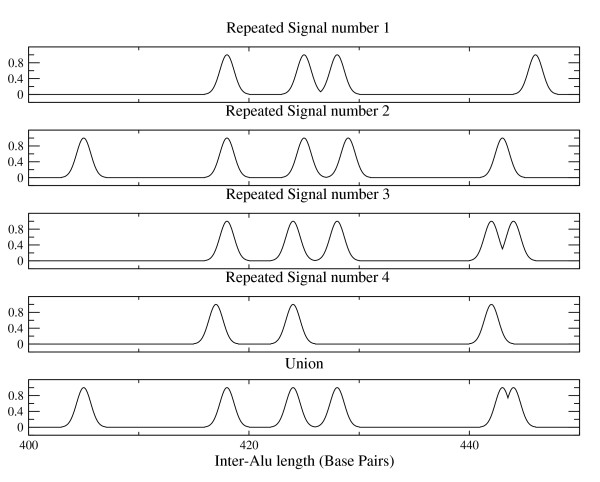
**Repeated signals**. Scheme of the union procedure for the 4 repeated signals; the peak in the union signals are obtained using an "or" procedure with an average on the peak position.

The lengths of inter-Alu PCR products range from 50 bp to 2000 bp (see Figure 1); we have chosen to restrict the analysis only to fragments up to 1000 bp interval since longer inter-alu fragments have low resolution in the experiments. Moreover the inter-Alu pattern turns out to be more robust and reproducible within this interval. The signals have been processed using a windowing cut-off, which suppresses the small peaks (approximately less than 10% of the local signal amplitude). The main reason to apply a local windowing cut-off procedure is that the PCR amplification characteristics depend on the DNA fragment length in capillary electrophoresis separation: indeed the original signals show regions with different mean peak amplitude, whose length is of order ≃ 300 bp. Then we choose a typical window of 250 bp width: this turns out to be a good compromise to have enough statistics (number of peaks) and signal regions with homogeneous characteristics for noise reduction. In order to extract the relevant information from the four repeated signals of each sample we adopted a "union procedure" based on the assumption that each peak of the filtered signals corresponds to a real inter-Alu sequence. This procedure reduces the experimental variability introduced by the PCR amplification. The union procedure uses a local alignment of the signals with a tolerance of 5 bp for each peak and produces a "union signal" using a logical OR function applied to the four signals; the final position of a peak is the average of the peaks positions in the four original signals (see Figure 2). The 5 bp tolerance is the result of measurement accuracy in the peak positions and it has been checked with repeated measures on the same samples. The peak position difference between twins in the same couple should be within this precision according to the assumption that they share the same genetic code, whereas we expect a statistical error less than 2 bp in the average peak position. Looking for genetic polymorphisms in the inter-Alu patterns we will use the twin signals to take into account such a variability. Each peak of the inter-Alu pattern is then normalized to a standard Gaussian function with unit amplitude and mean square value equal to 1 bp. Finally the resolution has been artificially increased to 32 points for each bp so that the final signals contain 1024 × 32 points: this choice allows for a "smooth" representation of the standard Gaussian peak suitable for the WPT.

## Results and Discussion

We have applied the WPT to the 6 union signals obtained from the three couples of twins and we have looked for the coefficients that discriminate among a fixed couple of twin and the others. The analysis of sample replicates reduces experimental variability mainly due to unpredictable errors due to the PCR reaction and to the electrophoretic separation. This reproduces the condition which is encountered in the routinary biological use of the inter Alu-PCR and other similar methodologies. In this case the variability between the twins of a given couple, that share the same genomic DNA sequence, can be explained by differences in DNA quality, purity, presence of contaminants and other unpredictable differences generated in the extraction and preparation of DNA samples (which could in principle partially depends from pre-existing biochemical/biological differences between the blood samples). The variability may appear as slightly different peak positions or different amplification degree of inter-Alu sequence that could produce non-detectable signals (peak absence in one twin).

**Figure 3:**
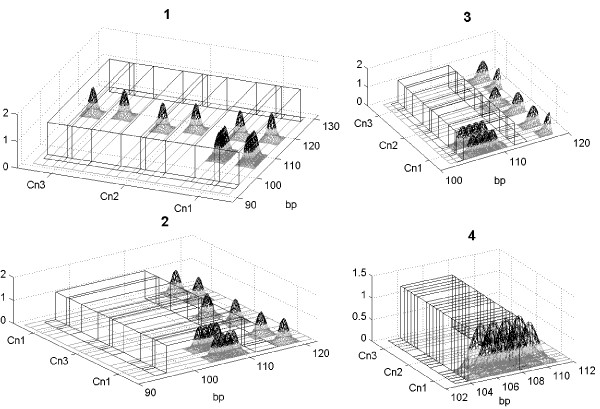
**Haar Walsh basis**. The normalized signals are shown together with the Haar-Walsh basis function that provides a maximal classification; the four figures refer to different scale levels in a decreasing order from fig. 1 to 4. On the *x*-axis we report the position in bp of the selected region; in the *y*-axis we report the six signals of the three twin couples (denoted by Cn1, Cn2 and Cn3). The amplitude of the normalized Gaussian peaks is measured along the *z*-axis. The Haar function that performs the signal classification is also drawn. The level 4 allows an optimal classification of the selected region of the first twins couple. The selected region is not a global classifying region since it does not distinguish between the signals of the second and the third twin couples.

The inter-Alu signals provided by the genetic analyzer discriminate ≃100 inter-Alu segments with a precision of 1 bp in the location. The WPT coefficients *c_ji _*are organized in a matrix *N *× 2*^N ^*, whose rows correspond to different scales: i.e. the *j *row is divided into 2*^j ^*blocks and each block contains the 2*^N-j ^*coefficients that define the wavelet translation along the scaled signal(see [25] for a mathematical presentation of WPT). We define {*c*_1*a*_}*_ji _*and {*c*_1b_}*_ji _*are the WPT coefficients of j level of the multiscale analysis for union signals of the first twin couple (the indexes *a *and *b *distinguish the two individuals). The difference

(1) $$\left|{\left\{c_{1a}-c_{1b}\right\}}_{ji}\right|$$

measures the variability of the given WPT coefficient for the signals of the first twin couple. A WPT coefficient {*c*_1*a*_}*_ji _*is selected if the variability (1) is significantly less than the variability of the same coefficient computed by comparing the signals of one member of the first couple and any other member of the other two twin couples. Therefore we introduce the following criterium for the selection process

(2) $$\underset{k=2,3;\;x,y\in \left\{a,b\right\}}{\min}\left|{\left\{c_{1x}-c_{ky}\right\}}_{ji}\right|-\left|{\left\{c_{1a}-c_{1b}\right\}}_{ji}\right|\geq \delta$$

where {*c_kx_*}*_ji _*denotes the coefficients produced by the union signal of the *x*-twin in the *k*-couple irrespectively of the member of the first twin couple. We recall our a priori assumption that the twins share the same genome and should have the same inter-Alu patterns. Therefore the observed differences are interpreted as the result of unavoidable variability in our experiments. The threshold *δ *has to be normalized with respect to the area of wavelet function support, associated to the coefficient *c_ji_*. Most of the selected coefficients analyze a common region of the original signals at different scale levels and can be ordered into a graph structure which allows to look for the coefficients that perform an optimal classification at the smallest decomposition scale. In Figure 3 we report an example of this procedure that detects the shortest regions of the signals where the patterns have the significant difference. To detect possible genetic polymorphisms the threshold value *δ *has been chosen equal to 1/3 in peak area unit, that allows to perform a classification between the twins couple based on a few number(≃ 10) of WPT coefficients. We defined "global classifying regions" the regions in the inter-Alu pattern that allow to distinguish simultaneously all the 3 couples of twins. These "global classifying regions" correspond to inter-Alu pattern regions that show reproducible differences among samples of different twin couples. Such regions may contain a peak in different position or a variable number of peaks, and they may be consequence of genetic polymorphisms in inter-Alu regions.

In order to relate the *δ *value with the effective differences in the inter-Alu patterns, we have to normalize the signals to the area of the support region of the wavelet function associated to the *c_ji _*coefficient. If, in the considered region, the union signals have a single peak, the criterium is satisfied when the peak position of different twin couples is shifted of 2 bp (at least) with respect to the measured difference between the peak position of the same twin couple. On the contrary if we are analyzing regions where several peaks are present, the criterium (2) takes into account the correlation among the peak positions in the signal and it is satisfied when the global difference between the patterns of different twin couples is more than 1/3 of the total signal area plus the experimental variability of the twin signals.

**Figure 4:**
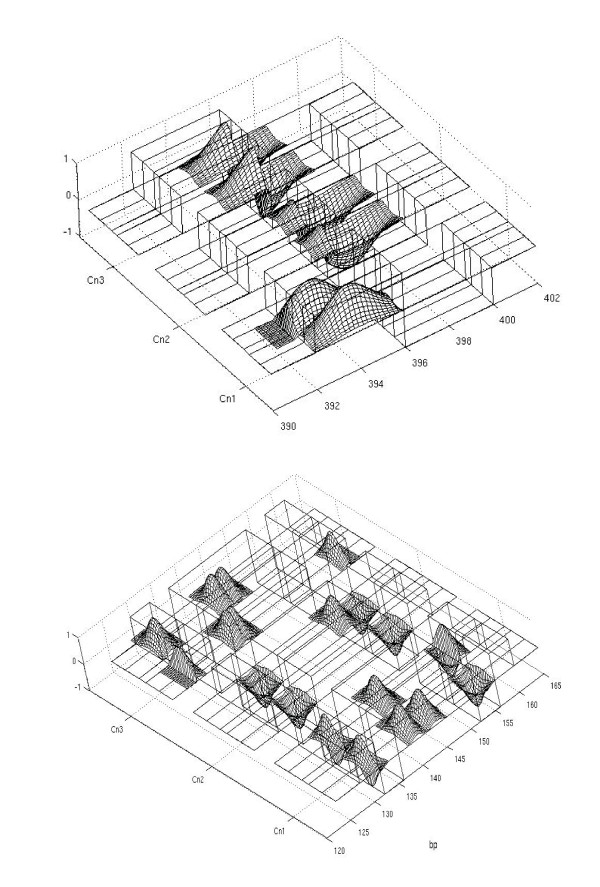
**Signal Classification**. Examples of global classifying regions. In the top picture the classification procedure based on WPT is applied on the interval 392-400 bp of the inter-Alu pattern. The classification is due to the presence of a single peak at different positions in the signals of the different twin couples Cn1, Cn2, Cn3, whereas it maintains the same position in the signals of a single twin couple. The wavelet function that performs the classification is positive in the interval 392-396 bp and negative in the interval 396-400 bp; therefore the convolution with the Gaussian peaks of the signal is the sum of positive and negative terms. The picture is obtained by multiplying the signals by the classifying wavelet function in order to illustrate the results of the WPT. The WPT coefficient is the sum of the positive and negative peak areas; therefore the WPT coefficient is positive for the couple Cn1, negative for the couple Cn2 and nearly zero for the couple Cn3 and a *K *value ≃1 is obtained in eq. (2). In the bottom picture the classification procedure is applied to a larger interval 120-160 bp. In such a case the WPT classification is due to the presence of a pattern of several peaks that have a significant variability among the signals of the different twin couples.

In Figure 4 we report two examples of global classifying regions of different size in the signals. We have repeated this analysis for the two different primers and the results are reported in Table 1 and Table 2. Most of the selected regions are narrow (less than 10 bp) with a value ≃1 in the criterium(2). These global classifying regions usually contain one or two variable peaks (see Figure 4 top), likely originating from a single locus carrying an insertion/deletion or a microsatellite (variable length of a short repeat) polymorphism. On the contrary, the global classifying regions wider than 10 bp (see Table 1) do often contain more than two variable peaks (see Figure 4 bottom). In such cases, two or more polymorphic loci are likely to be involved. While this "correlation" of different loci in the same global classifying region consists only by the fact that they originate inter-Alu PCR products of similar length, the crowding of many polymorphic peaks at specific length may be not casual, given the non-random genomic distribution of Alu repeats [17] and their non-random reciprocal distance and orientation [34]. Finally, we remark that the global classifying region (393-396) reported in Figure 4 corresponds to a polymorphic region (QM376-400) analyzed in our previous paper [10] using a different approach, which consists of a length polymorphism due to a dinucleotide microsatellite sequence located in an inter-Alu sequence on chromosome 1. The other possible polymorphisms pointed out by the WPT analysis were previously undetected.

**Table 1:** Global classifying regions obtained using the first marker (Tet fluorochrome)

Couple 1		Couple 2		Couple 3	
**Region(bp)**	***K***	**Region(bp)**	***K***	**Region(bp)**	***K***

129-160	0.99	129-160	0.98	129-160	1.91
197-200	0.45	197-200	0.45	197-200	0.57
289-296	0.41	289-296	0.57	293-296	0.44
329-336	0.73	329-336	0.69	329-336	0.82
393-396	0.94	393-400	1.01	397-400	0.95
529-536	0.82	529-532	0.52	529-532	0.43
609-612	0.38	609-612	0.44	609-616	0.89
641-648	0.81	641-656	0.65	641-656	0.76

The first column refers to the bp-length of the selected inter-Alu fragments, whereas the second column gives the corresponding *K *values of the criterium (2) in peak area units that allow the classification. We report results for the 3 twin couples.

**Table 2:** Global classifying regions obtained using the second marker (Fam fluorochrome)

Couple 1		Couple 2		Couple 3	
**Region(bp)**	***K***	**Region(bp)**	***K***	**Region(bp)**	***K***

65-80	0.71	65-96	0.74	65-96	0.79
109-128	1.64	113-128	0.62	97-128	1.57
353-360	1.61	353-384	1.05	353-360	1.14
465-480	1.45	465-468	0.77	465-468	0.71
833-896	2.21	849-896	1.70	849-896	2.94

The structure of the table is the same as table 1. At variance with table 1, we observe that the detected regions have a large bp amplitude.

## Conclusions

The WPT algorithm based on the Haar-Walsh wavelet allows for a non-supervised pattern classification of inter-ALU signals obtained using a genetic analyzer. We tested the algorithm on inter-Alu PCR patterns of three couples of monozygotic twins. The pattern obtained using this genomic fingerprinting methodology, based on inter-Alu PCR and capillary electrophoresis, is very complex and results in more than 100 peaks. Such a number of inter-Alu amplification products is compatible with the observed distribution of Alu sequences along the human genome, characterized by a great variability from the 10% average Alu density, and by the presence of a limited number of genomic regions having an exceptionally high Alu density [17]. The subjective evaluation of the patterns is hampered by the presence of some non-reproducible peaks that should be excluded from the analysis [10]. The identification of non-reproducible peaks is usually accomplished by comparing different experimental replicates of each sample. However, in our assay we used not only four replicates for each sample, but even DNA samples from monozygotic twins. In this way, we had for each sample a "super-replicate" (the DNA from the other member of the twin couple) that allowed for a more reliable identification of those regions of the pattern whose variability was due to unpredictable experimental variations, and not to differences in the genomic DNA. The application of the WPT algorithm detected 13 polymorphic regions of the inter-Alu pattern; one of them corresponded to the previously detected (by "visual" analysis) QM376-400 region [10], whereas the others were previously undetected. Albeit a precise comparison of the present method with the results obtained by analyzing the Alu-PCR pattern "by eye" (the commonly adopted procedure) is diffcult due to the subjective and operator-dependent nature of this procedure, the present method promises a better sensitivity, given that 13 global classifying regions have been detected in the present work (in three couples of twins) vs. 3 polymorphic regions detected "by eye" in a previous study [10] on a larger set of samples. We have to remark that the obtained results have some important limitations. In particular, we are not in condition to give any estimate of the power and false positive rate of the present application of WPT algorithm in the detection of Alu-PCR polymorphisms, since it does not exist any widely-accepted data set that can be used as a standard test case. This would require the cloning, sequencing and characterization of a large number of polymorphic inter-Alu PCR products, with an economic and research effort far beyond the scope of this work. While we are aware that such a limitation can hamper the objective evaluation of the performance of the present software and that this issue deserves to be addressed in future papers, it should be noted that the same limitation is also true for the traditional "by eye" approach (ignoring the power and false positive rate of this methodology). On the whole, the advantages of the application of the WPT algorithm with respect to the "visual", subjective inspection of electrophoretic patterns can be summarized as follows:

a) a rapid, computer-assisted detection of variable peaks;

b) an automated comparison of different replicates of the same sample, and an automatic "extraction" of reproducible signals;

c) a better sensitivity, with the ability to detect an higher number of polymorphic regions.

Moreover we remark that, albeit we developed an algorithm specifically optimized to analyze inter-Alu PCR patterns, the method is theoretically applicable to whatever fingerprint-type pattern obtained analyzing anonymous DNA fragments through capillary electrophoresis, and could be usefully applied on a wide range of fingerprint-type methodologies. It is important to note that, recently, new high-throughput methods based on DNA sequencing [35] and on TIP-chip microarray analysis [36,37] have been presented, aimed to perform a locus by locus detection of Alu mutation/polymorphisms on the whole genome: the first results obtained with these methodologies [35,37] have begun to clarify and to point out the importance of the mutagenesis mediated by Alu sequences and other retrotransposons in human genome variation and in various disease conditions. However, for their inherent complexity and high cost, these high-throughput methodologies are not likely to become (at least in the next few years) a substitute for inter-Alu PCR in all those situations in which limited availability of time or budget could be a constraint (for example, for diagnostic examination of disease states in which the importance of Alu-associated genetic variation has been found). The availability of a computer method capable to speed-up, simplify and standardize the analysis of inter-Alu PCR patterns will be a valuable aid for a routine use of the inter-Alu analysis.

## 1 Authors' contributions

MC performed the inter-Alu PCR on the biological samples and participated in designing the study and drafting the manuscript, MN participated in implementing the WPT algorithm and performed the WPT analysis on the inter-Alu patterns, AB designed the study and participated in implementing the WPT algorithm and drafting the manuscript, CF designed the study and collected the biological samples. All authors read and approved the final manuscript.

## References

[B1] Williams JG Kubelik AR Livak KJ Rafalski JA Tingey SV DNA polymorphisms amplified by arbitrary primers are useful as genetic markers Nucleic Acids Res 1990 18 6531 6535 10.1093/nar/18.22.6531 1979162 PMC332606

[B2] Welsh J M M Fingerprinting genomes using PCR with arbitrary primers Nucleic Acids Res 1990 18 7213 7218 10.1093/nar/18.24.7213 2259619 PMC332855

[B3] Zietkiewicz E Rafalski A Labuda D Genome fingerprinting by simple sequence repeat (SSR)-anchored polymerase chain reaction amplification Genomics 1994 20 176 183 10.1006/geno.1994.1151 8020964

[B4] Liang P Pardee AB Differential display of eukaryotic messenger RNA by means of the polymerase chain reaction Science 1992 257 967 971 10.1126/science.1354393 1354393

[B5] Vos P Hogers R Bleeker M Reijans M van de Lee T Hornes M Frijters A Pot J Peleman J Kuiper M AFLP: a new technique for DNA fingerprinting Nucleic Acids Res 1995 18 7213 7218 10.1093/nar/23.21.4407 PMC307397 7501463

[B6] Nelson DL Ledbetter SA Corbo L Victoria MF Ramirez-Solis R Webster TD Ledbetter DH Caskey CT Alu polymerase chain reaction: a method for rapid isolation of human-specific sequences from complex DNA sources Proc Natl Acad Sci USA 1989 86 6686 6690 10.1073/pnas.86.17.6686 2771952 PMC297910

[B7] Lavanya GR Srivastava J Ranade SA Molecular assessment of genetic diversity in mung bean germplasm J Genet 2008 87 65 74 10.1007/s12041-008-0009-3 18560176

[B8] Shifat R Begum A Khan H Use of RAPD fingerprinting for discriminating two populations of Hilsa shad (Tenualosa ilisha Ham.) from inland rivers of Bangladesh J Biochem Mol Biol 2003 36 462 467 14536029 10.5483/bmbrep.2003.36.5.462

[B9] Johnson EL Zhang D Emche SD Inter- and Intra-specific Variation among Five Erythroxylum Taxa Assessed by AFLP Ann Bot (Lond) 2005 95 601 608 10.1093/aob/mci062 PMC4246853 15650009

[B10] Bonafè M Cardelli M Marchegiani F Cavallone L Giovagnetti S Olivieri F Lisa R Pieri C Franceschi C Increase of homozygosity in centenarians revealed by a new inter-Alu PCR technique Experimental Gerontology 2001 36 1063 1073 11404051 10.1016/s0531-5565(01)00112-7

[B11] Sinnett D Deragon JM Simard LR Labuda D Alumorphs human DNA polymorphisms detected by polymerase chain reaction using Alu-specific primers Genomics 1990 7 331 334 10.1016/0888-7543(90)90166-R 1973138

[B12] Cardelli M Alu PCR Methods Mol Biol 2011 687 221 229 full_text 20967611 10.1007/978-1-60761-944-4_15

[B13] Jarnik M Tang JQ Korab-Laskowska M Zietkiewicz E Cardinal G Gorska-Flipot I Sinnett D Labuda D Overall informativity, OI, in DNA polymorphisms revealed by inter-Alu PCR: detection of genomic rearrangements Genomics 1996 36 388 398 10.1006/geno.1996.0483 8884261

[B14] Lander ES Linton L Birren B Nusbaum C Zody M Baldwin J Devon K Dewar K Doyle M FitzHugh W Initial sequencing and analysis of the human genome Nature 2001 409 860 921 10.1038/35057062 11237011

[B15] Zietkiewicz E Labuda M Sinnett D Glorieux FH Labuda D Linkage mapping by simultaneous screening of multiple polymorphic loci using Alu oligonucleotide-directed PCR Proc Natl Acad Sci USA 1992 89 8448 8451 10.1073/pnas.89.18.8448 1528850 PMC49937

[B16] Mighell AJ Markham AF Robinson PA Alu sequences FEBS Lett 1997 417 1 5 10.1016/S0014-5793(97)01259-3 9395063

[B17] Cardelli M Marchegiani F Cavallone L Olivieri F Giovagnetti S Mugianesi E Moresi R Lisa R Franceschi C A polymorphism of the YTHDF2 gene (1p35) located in an Alu-rich genomic domain is associated with human longevity J Gerontol A Biol Sci Med Sci 2006 61 547 556 16799135 10.1093/gerona/61.6.547

[B18] Krajinovic M Richer C Labuda D Sinnett D Detection of a mutator phenotype in cancer cells by inter-Alu polymerase chain reaction Cancer Res 1996 56 2733 2737 8665504

[B19] Furmaga WB Cole SR Tsongalis GJ The use of Alu-PCR to distinguish between typical pulmonary carcinoids versus classic midgut carcinoids Int J Oncol 2004 24 223 226 14654961 10.3892/ijo.24.1.223

[B20] McKie AB Iwamura T Y LH Hollingsworth MA Lemoine NR Alu-polymerase chain reaction genomic fingerprinting technique identifies multiple genetic loci associated with pancreatic tumourigenesis Genes Chromosomes Cancer 1997 18 30 41 10.1002/(SICI)1098-2264(199701)18:1<30::AID-GCC4>3.0.CO;2-2 8993978

[B21] Furmaga WB Ryan JL Coleman SR Tsongalis GJ Alu profiling of primary and metastatic non-small cell lung cancer Exp Mol Pathol 2003 74 224 229 10.1016/S0014-4800(03)00016-9 12782008

[B22] Srivastava T Seth A Datta K Chosdol K Chattopadhyay P Sinha S PCR detects high frequency of genetic alterations in glioma cells exposed to sub-lethal cisplatin Int J Cancer 2005 117 683 689 10.1002/ijc.21057 15912534

[B23] Pal A Srivastava T Sharma MK Mehndiratta M Das P Sinha S Chattopadhyay P Aberrant methylation and associated transcriptional mobilization of Alu elements contributes to genomic instability in hypoxia J Cell Mol Medpublished online Jun 2009 19508390 10.1111/j.1582-4934.2009.00792.x PMC4373486

[B24] Moyzis RK Torney DC Meyne J Buckingham JM Wu JR Burks C Sirotkin KM Goad WB The distribution of interspersed repetitive DNA sequences in the human genome Genomics 1989 4 273 289 10.1016/0888-7543(89)90331-5 2714792

[B25] Jensen A la Cour-Harbo A Ripples in Mathematics: The Discrete Wavelet Transform 2001 New York: Springer-Verlag

[B26] Klevecz RR Dynamic architecture of the yeast cell cycle uncovered by wavelet decomposition of expression microarray data Funct. Integr. Genomics 2000 1 186 192 10.1007/s101420000027 11793236

[B27] Wang J Ma JZ Li MD Normalization of cDNA Microarray Data Using Wavelet Regressions Combinatorial Chemistry & High Throughput Screening 2004 7 783 791 10.2174/1386207043328274 15578940

[B28] Wen SY Zhang CT Identification of isochore boundaries in the human genome using the technique of wavelet multiresolution analysis Biochemical and Biophysical Research Communications 2003 311 215 222 10.1016/j.bbrc.2003.09.198 14575716

[B29] Lio P Vannucci M Finding pathogenicity islands and gene transfer events in genome data Bioinformatics 2000 16 10 932 940 10.1093/bioinformatics/16.10.932 11120683

[B30] Lio P Wavelets in bioinformatics and computational biology: state of art and perspectives Bioinformatics 2003 19 2 9 10.1093/bioinformatics/19.1.2 12499286

[B31] Coifman R Wickerhauser MV Entropy-Based Algorithms for Best Basis Selection IEEE Transactions on Information Theory 1992 38 713 718 10.1109/18.119732

[B32] Saito N Coifman R Improved local discriminant bases using empirical probability density estimation Proceedings of Statistical Computing 1996

[B33] Daubechies I Ten lectures on wavelets 1992 Philadelphia: Society for Industrial and Applied Mathematics

[B34] Stenger JE Lobachev KS Gordenin D Darden TA J J Resnick MA Biased distribution of inverted and direct Alus in the human genome: implications for insertion, exclusion, and genome stability Genome Res 2001 11 12 27 10.1101/gr.158801 11156612

[B35] Iskow RC McCabe MT Mills RE Torene S Pittard WS Neuwald AF G VME Vertino PM Devine SE Natural mutagenesis of human genomes by endogenous retrotransposons Cell 2010 141 1253 1261 10.1016/j.cell.2010.05.020 20603005 PMC2943760

[B36] Cardelli M Marchegiani F Franceschi C Lattanzio F Provinciali M Alu insertion site profiling in the human genome (abstract) New Biotechnology 2010 27 S38 10.1016/j.nbt.2010.01.050

[B37] Huang CR Schneider AM Lu Y Niranjan T Shen P A M P SJ Valle D Civin CI Wang T Wheelan SJ Ji H Boeke JD Burns KH Mobile interspersed repeats are major structural variants in the human genome Cell 2010 141 1171 1182 10.1016/j.cell.2010.05.026 20602999 PMC2943426

